# Electrical lysis of cells for detergent-free droplet assays

**DOI:** 10.1063/1.4944742

**Published:** 2016-03-22

**Authors:** N. de Lange, T. M. Tran, A. R. Abate

**Affiliations:** 1 Department of Physical Chemistry and Soft Matter, Wageningen University, Dreijenplein 6, 6703 HB, Wageningen, The Netherlands; 2 Bioengineering and Therapeutic Sciences, University of California San Francisco, San Francisco, California 14958, USA

## Abstract

Efficient lysis is critical when analyzing single cells in microfluidic droplets, but existing methods utilize detergents that can interfere with the assays to be performed. We demonstrate robust cell lysis without the use of detergents or other chemicals. In our method, cells are exposed to electric field immediately before encapsulation in droplets, resulting in cell lysis. We characterize lysis efficiency as a function of control parameters and demonstrate compatibility with enzymatic assays by measuring the catalysis of β-glucosidase, an important cellulase used in the conversion of biomass to biofuel. Our method enables assays in microfluidic droplets that are incompatible with detergents.

## I. INTRODUCTION

Cellular heterogeneity is important in a variety of biological systems, from providing robustness to evolutionary stresses to enabling effective immune responses against diverse threats.^1–3^ Because the heterogeneity exists at the level of single cells, studying these systems requires methods for high-throughput single cell analysis. Flow cytometry enables the detection, characterization, and sorting of single cells at throughputs of >1000 per second,^4^ allowing large populations to be screened in hours; however, it is limited by its dependence on affinity reagents that specifically label the target cell so that it can be detected within a mixed population. Droplet microfluidics breaks through this barrier by allowing single cells to be analyzed using soluble assays, such as enzyme catalysis,^5^ detection of secreted products,^6–8^ or presence of unique nucleic acid sequences.^9,10^ The devices achieve this using tools for rapidly generating,^11^ merging,^12^ injecting,^13^ and sorting droplets^14^ for applications including single-cell sequencing,^15,16^ directed evolution,^17,18^ and drug screening.^19^

When using droplet microfluidics for high-throughput single cell analysis, cellular lysis is essential to provide access to cell contents, such as specific small molecules, proteins, or nucleic acids.^10,20–22^ Lysis of cells can be achieved using chemical (e.g., detergents),^9,10^ optical (e.g., pulsed laser),^23^ mechanical (e.g., nanoknives),^24^ acoustic (e.g., sonication),^25^ or electrical^26^ techniques. In droplet microfluidics, robust lysis is most commonly achieved using proteases and detergents that digest proteins and solubilize cellular lipids.^9,10^ However, proteases can digest the enzymes necessary for assays, while detergents are difficult to remove from droplets once added and can interfere with important interactions between molecules. Consequently, when using these components to lyse cells, compatible assays must be carefully selected and, even then, the assay may be influenced by their presence. For example, detergents commonly used for lysing cells can perturb the stability and activity of enzymes so that measurements performed with detergents often do not agree with the ones performed detergent-free.^27^ To enable greater flexibility when choosing assays with which to analyze single cells in microfluidic droplets, new, chemical-free methods are needed for lysing cells.

In this paper, we present a simple, chemical-free method for lysing cells compatible with nearly any droplet assay. Previous work has demonstrated the ability to generate pores in cell membranes by applying electric fields, a method known as electroporation.^28^ This is often used to introduce components into cells that cannot normally pass the membrane, such as nucleic acids and certain small molecules, and can be performed with microfluidics.^29–37^ Here, we extend this concept to lyse cells by applying an electric field immediately before merging the cell stream with lysozyme and encapsulating the mixture in droplets. Pulses above the electroporation threshold have been shown to generate pores in the cell membrane that persist for seconds to minutes after the field is removed, providing ample time for lysosome to diffuse into the cell and inter-membrane space and digest the cell wall, ultimately culminating in lysis. As we demonstrate, with lysozyme alone, lysis is poor, whereas when the electric field pulse is added, lysis efficiencies >90% can be achieved. We characterize the dependence of lysis efficiency on multiple parameters and use the method to measure the activity of β-glucosidase, a cellulase used in biomass deconstruction. Our lysis approach broadens the types of assays that can be used in droplet microfluidics without sacrificing lysis efficiency.

## II. MATERIALS AND METHODS

### A. Microfluidic fabrication

Photoresist (SU-8 3010) is spin coated onto silicon wafers and cross-linked in the pattern of the microfluidic device using photo-masks and ultra-violet light exposure, followed by the development of the master and baking. Poly(dimethylsiloxane) (PDMS) replicas of the device^38^ are cast by pouring 11:1 ratio of base to curing agent (Sylgard 184, Dow Chemical, MI, USA) and baking at 80 °C for 1 h. The replicas are extracted from the wafer with a scalpel and access holes are punched with a 0.75 mm biopsy punch. The device is rinsed with isopropanol and bonded to a glass slide using oxygen plasma treatment. All devices are treated with Aquapel (PPG Industries) and baked for 30 min at 80 °C to render them hydrophobic for water-in-fluorinated oil emulsification. The height of the fabricated channels is 20 *μ*m.

### B. Green fluorescent protein (GFP) assay

*GFP* is cloned into the pET-22b vector and transformed into electrocompetent BL21(DE3) *Escherichia coli* (Lucigen). Expression recovery media (Lucigen) is used to recover transformed cells by incubating at 37 °C for 1 h. A glycerol stock of the library is made by combining cell media with 50% glycerol and stored at −80 °C until use. 5 ml MagicMedia (Invitrogen) expression culture is inoculated using the glycerol stock library and incubated overnight at 37 °C, followed by pelleting and re-suspension in the assay buffer (100 mM tris(hydroxymethyl)aminomethane (Tris), pH 7.5). The cell solution is further diluted in the assay buffer to achieve an OD600 of 0.025. The lysozyme solution is produced by diluting rLysozyme (Novagen) to a final concentration of 60 KU/ml in 100 mM Tris, pH 7.5. In the experiments, multiple parameters are varied including aqueous flow rates (50 and 100 *μ*l/h), oil flow rates (200, 300, 400, and 600 *μ*l/h), salt concentrations in the assay buffers (0, 50, 100, 200, and 500 mM NaCl), electroporation channel dimensions (30 × 5000, 60 × 2500; 120 × 1250; and 240 × 625 *μ*m), and the presence of lysozyme.

### C. β-Glucosidase assay

A *BGL3* gene insert is cloned into the pET-22b vector and transformed into electrocompetent BL21(DE3) *E. coli* (Lucigen). Expression recovery media (Lucigen) are used to recover the transformed cells by incubating at 37 °C for 1 h and cell plating. Once colonies have developed, they are stored at 4 °C until use. A single colony is used to inoculate a 5 ml MagicMedia (Invitrogen) expression culture. The culture is incubated 37 °C overnight, pelleted, and re-suspended in the assay buffer (100 mM potassium phosphate, pH 7.2). The cell solution is further diluted in assay buffer to a final cell suspension of OD600 of 0.025 in combination with 1 *μ*g/ml DAPI (4′,6-Diamidino-2-Phenylindole, Dihydrochloride) (Life Technologies). The lysis buffer for detergent based lysis consists of 0.6× BugBuster (Novagen), 60 KU/ml rLysozyme (Novagen), and 200 *μ*M fluorescein di-(β-d-glucopyranoside) (Sigma) in 100 mM potassium phosphate, pH 7.

### D. Microfluidic device operation

**FIG. 1.**
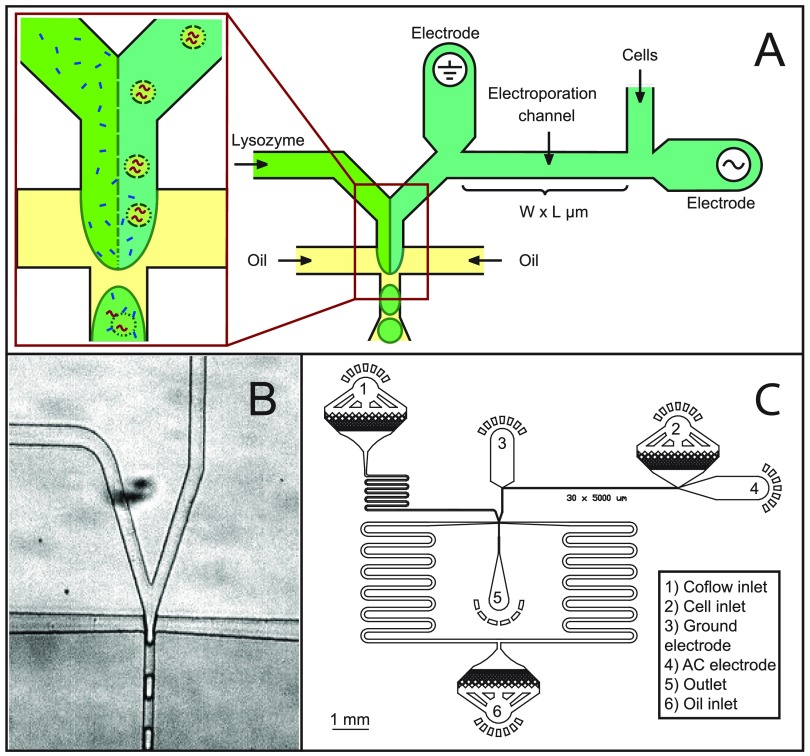
Schematic representation of electrical lysis for droplet screening. (a) Schematic of the electrical lysis part and co-flow droplet generation part of the microfluidic device. (b) Actual image of the droplet generation part. (c) A to-scale view of the whole electrical lysis device with electroporation channel dimensions of 30 *μ*m × 5000 *μ*m.

Microdroplets are generated using a co-flow electroporation droplet maker (Figure 1) consisting of an inlet for the cell suspension and a co-flow inlet for the lysis buffer, followed by a cross-junction into which oil is introduced (HFE-7500 fluorinated oil with 2 wt. % fluorinated surfactant, RAN Technologies) to generate the droplets. Cell densities are controlled to yield ∼1 cell per 10 drops. The generated droplets (∼27–29 *μ*m in diameter) are collected into 1.5 ml Eppendorf tubes and incubated at room temperature for 5–10 min prior to imaging. The cell stream flows through an electrified channel to initiate electrically induced cell lysis before merging with the lysis buffer stream. To generate an electric field in the electroporation channel, electrodes consisting of lead-free solder material (Super Solder, 0.8 mm alloy no. 60) are connected to an AC amplifier (JKL Components Corp., 289-1170-ND) powered by a DC supply. The voltage is applied as a 34 kHz sine wave with an amplitude of 0 to 1300 V, from which we calculate the field applied to the cells by modeling the electrical resistivity of the conducting electrolyte-filled channels through which the cells pass as they enter the droplet generator.^29,30^ We estimate the maximum amplitude of the currents to be ∼12 mA. Since the electrodes are in contact with the aqueous phase carrying the cells, it is possible that electrochemical products generated by the flow of current may end up in the encapsulating droplets; however, we do not directly observe any such products nor do the assays we perform seem to be perturbed.

### E. Lysis quantification

Droplets are loaded into Countess cell counting chamber slides (Life Technologies), and single layers are imaged using an inverted fluorescence microscope (EVOS^®^ FL Auto Imaging System, Life Technologies) in bright field and fluorescence modes with 470/22 nm wavelength excitation and 510/42 nm emission (GFP channel). Droplets for the β-glucosidase experiment are additionally imaged with 357/44 nm excitation and 447/60 nm emission to visualize DAPI, a DNA stain which we used to identify drops containing cells.

### F. Image analysis

Bright field and fluorescence images are analyzed using ImageJ by selecting a threshold such that droplets or cells appear as disconnected areas on a dark background. Areas of 18–400 *μ*m^2^ correspond to small, unlysed cells with localized fluorescence, while ones with 400–1000 *μ*m^2^ correspond to lysed cells in which the cell lysate diffuses into the encapsulating droplet, making the droplet diffusely fluorescent. Lysis efficiency is calculated as the number of lysed cells divided by the sum of lysed and unlysed cells.

## III. RESULTS AND DISCUSSION

A principal advantage of droplet microfluidics is the extremely high throughput with which individual cells can be analyzed using soluble assays. Leveraging this advantage requires a robust method for lysing cells that minimally interferes with the assays to be performed. Our method for accomplishing this is to flow the cells through a channel with high electric field before encapsulating them in droplets. This pulses the cells with electric field, where the duration of the pulse is determined by the flow rate and the amplitude by channel geometry and voltage. We also include lysozyme, an enzyme that digests bacterial cell walls but minimally interferes with most assays, via a second channel that intersects with the cell-containing channel at the droplet maker, as shown in Fig. 1(a). An image of the droplet generator is provided in Fig. 1(b) and a to-scale schematic of the entire device in Fig. 1(c). As a cell passes through the device, it first flows through the electric field channel; the electric field is sufficient to electroporate the cells, but they remain intact as cell bodies. In addition, the Péclet number relating the ratio of advective to diffusive transport is ∼10 000, indicating that as the cells travel through the electrification channel, they remain localized in their streamlines; this ensures that each cell's lysate is encapsulated into a single droplet.

**FIG. 2.**
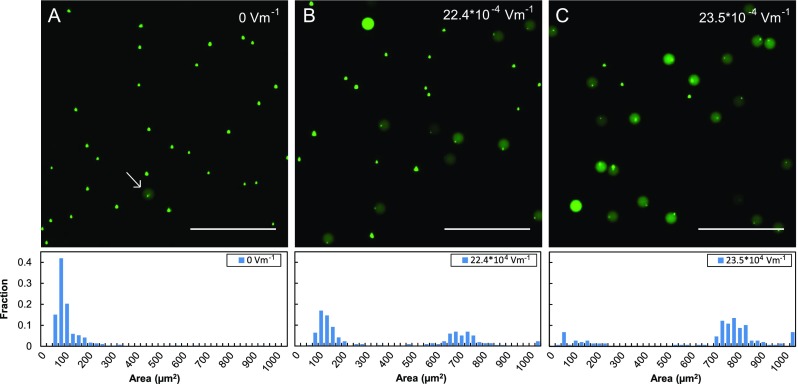
Example of the effects of electrical lysis on encapsulated *E. coli* cells expressing GFP. Green fluorescence pictures of the cells taken when (a) no electric field was applied, (b) 22.4 × 10^4 ^V m^−1^ was applied, and (c) 23.5 × 10^4 ^V m^−1^ was applied. As the electric field increases, the fraction of lysed cells increases, which is quantitatively shown in the histogram below the image. The scale bar in the images represents 100 *μ*m.

To investigate the ability of this technique to lyse bacterial cells, we test the approach with *E. coli* engineered to express GFP. We flow the cells into the device at 100 *μ*l/h using a 30 *μ*m × 5000 *μ*m × 20 *μ*m electroporation channel, exposing them to the electric field for ∼100 ms prior to encapsulation. The parallel co-flow stream contains the lysis comprising lysozyme introduced at the same flow rate. After passing through the device and being encapsulated in the droplets, unlysed *E. coli* appear as compact, bright puncta a few microns in diameter, while lysed cells appear as diffuse green fluorescence filling the encapsulating droplet. When no field is applied, 99% of cells remain unlysed, as shown in Fig. 2(a). By contrast, when we increase the electric field to 22.4 × 10^4 ^V m^−1^ roughly half the cells lyse (Fig. 2(b)) while at 23.5 × 10^4 ^V m^−1^, ∼70% lyse (Fig. 2(c)). These results can be rationalized based on the membrane structure of *E. coli*, which have an inner and outer membrane separated by a cell wall. Lysis occurs when the bacterial membrane is irreversibly permeabilized. While electroporation can create pores in the cell membrane, bacteria also possess a cell wall that protects their inner membrane. Lysozyme breaks down this cell wall to enhance lysis efficiency.^39,40^ Indeed, lysozyme lyses cells slowly over time, which is why it must be added immediately before encapsulation using co-flow droplet generation; if it was added to the cell suspension long before encapsulation, pre-lysis in the syringe would allow the lysates of different cells to mix, precluding the execution of pure single cell assays. Co-flow droplet generation enables this because cell and lysozyme solutions do not mix until they are encapsulated in the droplets, due to laminar flow conditions.^41^

The electric fields we apply are comparable to what is used in the food industry (20–40 kV/cm) to lyse microbes for food preservation. Multiple studies have investigated enzyme inactivation by exposure to such electric fields and have found, generally, that enzymes are more resistant to electric fields than microbes;^42,43^ above a threshold field, enzyme activity can be reduced, though the behavior depends on the enzyme under consideration.^44^

**FIG. 3.**
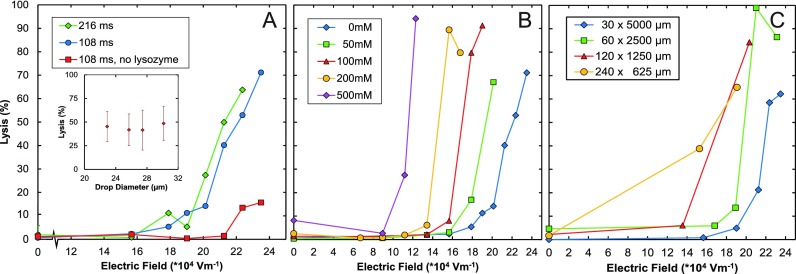
Dependence of lysis efficiency for different control parameters. (a) Lysis efficiency as a function of voltage for different times of field exposure, with and without lysozyme. (Inset) Lysis percentage versus drop diameter showing little dependence, achieved by varying the oil flow rate. (b) Lysis efficiency as a function of voltage for different salt concentrations showing substantial dependence. Highest condition is equivalent to saltwater of the ocean. (c) Lysis efficiency as a function of voltage for different channel dimensions, holding the time of field exposure for the cell constant.

Achieving efficient cell lysis requires knowledge of the parameters that most greatly impact the electroporation process. To investigate this, we systematically vary parameters and observe the impact on lysis efficiency (Fig. 3). To measure the lytic effect for lysozyme, each series of experiments includes a control in which we do not apply an electric field; the lysis efficiencies of these experiments are consistently close to zero, which implies that on the timescale of our experiments, lysozyme alone is ineffective for efficient lysis. When we apply the electric field but do not include lysozyme, we obtain lysis efficiencies of <20%, shown by the red points in Fig. 3(a). Combining both lysozyme and electrical lysis improves the lysis efficiency significantly, resulting in lysis efficiencies up to 70% with these conditions.

An important parameter is the time the cell is exposed to the electric field, since this influences the duration that the pores are maintained through which lysozyme can enter. Millisecond pulses in the range of 1 kV/cm, comparable to what we apply, can yield pores with lifetimes of minutes, providing ample time for lysozyme to diffuse into the inter-membrane space,^28^ where it can digest the cell wall. To vary this parameter, we adjust the flow rate of the cell solution using the 30 × 5000 *μ*m device (Figure 3(a)). When lysozyme is present, the fraction of cells lysed strongly increases with amplitude of the electric field, but does not depend strongly on the duration of field exposure for the range tested, as shown by the green and blue points in Fig. 3(a). This indicates that even the shortest pulse duration is able to generate pores sufficient for cell lysis. To confirm that these results do not depend on the size of the encapsulating droplets, which decreases as we reduce flow rate to increase field exposure time, we perform a second series of experiments holding exposure time and field strength constant, varying droplet size by adjusting carrier oil flow rate. However, again, there appears to be little dependence of lysis efficiency on this parameter, as shown in Fig. 3(a), inset.

Another parameter that impacts lysis efficiency is the conductivity of the buffer: Holding field amplitude constant, higher buffer conductivity increases electric current, which can, in turn, impact lysis efficiency. To investigate this, we vary buffer conductivity by adjusting NaCl concentration while holding other parameters constant (Fig. 3(b)). Similar to previous experiments, we find that, generally, the fraction of lysed cells increases strongly with electric field, with a range of low fields in which very little lysis is observed, followed by an abrupt increase in lysis above a threshold value. We also find that lysis efficiency depends on the conductivity of the buffer, with high conductivity buffers leading to cell lysis at lower fields than low conductivity buffers, as shown in Fig. 3(b). Hence, while salt concentration is an important parameter because it impacts the conductivity of the solution, high or low salt alone is not able to lyse the cells over the timescales we have tested, as shown by the low lysis efficiencies achieved for zero applied field.

The field in the electroporation channel depends on the applied voltage and resistivity of the path connecting the positive and ground electrodes, which, in turn, depends on the length of the connecting channel. To investigate this, we vary the lengths and widths of these channels to maintain the time that the cells flow through the channel constant (Fig. 3(c)). As expected, there is only a weak dependence on the shape of the electroporation channel. Importantly, however, the device with wider, shorter channels achieves the needed electric fields to lyse cells at lower voltages; in addition, its hydrodynamic resistance is also lower, lowering the input pressure of the device. Hence, if low operating pressures and voltages are desired, a short, wide electroporation channel is preferable to a long, narrow one.

Our results indicate that there is a threshold field above which electrically induced lysis becomes efficient and this field is lower when highly conductive buffers are used. Lysozyme, while ineffective on its own, greatly enhances lysis efficiency when used with electroporation. The time of electroporation, geometry of the channel, or size of the encapsulating droplets also affect lysis to varying degrees, as summarized in Fig. 3.

**FIG. 4.**
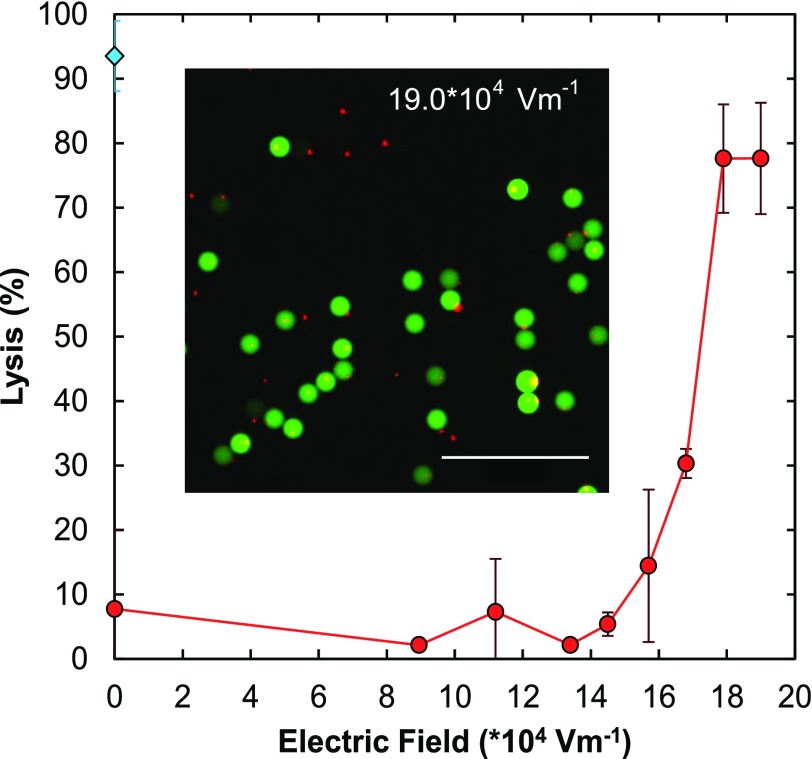
Application of electrical lysis for an enzymatic assay. Plot of efficiency of lysis as a function of electric field for *E. coli* cells expressing the enzyme β-glucosidase (red curve). The substrate for the enzyme is included in the droplet by the second inlet of the coflow running parallel to the cells downstream of the electrical lysis region. A fluorescence image of the resulting droplets is shown in the inset where the small red dots correspond to the DAPI stained cells and the larger green circles correspond to the encapsulated droplets. The green fluorescence is the product of catalysis of the fluorigenic substrate of β-glucosidase.

An important example of droplet-based microfluidic screening that relies on robust cell lysis is measuring enzyme catalysis at the single cell level, both for characterizing enzyme activity or enhancing it through droplet-based directed evolution.^17,18^ Chemical lysis is often undesirable because chemicals can interfere with the catalysis assay, whereas our electrical technique adds no interfering chemicals. To demonstrate this, we measure the activities of β-glucosidase, an enzyme important in the conversion of biomass into biofuel, expressed in *E. coli* (Fig. 4). We flow the cells through the 30 × 5000 *μ*m electroporation channel at 100 *μ*l/h, dispersing them in potassium phosphate buffer, pH 7.2. When the cells are lysed, the enzyme expressed within them leaks into the encapsulating droplet where it catalyzes the breakdown of the substrate producing a fluorescent signal, as shown by the diffusely green-fluorescent droplets inset into Fig. 4. In agreement with our previous studies utilizing GFP, we find that the percentage of lysed cells increases with electric field strength, with a threshold field of ∼14 × 10^4 ^V m^−1^. To confirm that the observed catalysis results from cells, we stain the cells with DAPI prior to encapsulating them, so that they appear as small red dots in the image (Fig. 4). While there are indeed a small number (∼20%) of encapsulated cells with no β-glucosidase in the drops, the majority lyse releasing β-glucosidase to catalyze the reaction. As a comparison, we repeat the experiment using a chemical lysing agent, BugBuster, to lyse the cells (blue point, 0 electric field, Fig. 4). While BugBuster outperforms electrical lysis for the conditions tested, it only does so by ∼10%, and optimization of buffer conductivity and channel dimensions may enable comparable lysis efficiency. Moreover, BugBuster, which contains detergents, can interfere with sensitive catalysis assays.

## IV. CONCLUSIONS

We have developed a robust method for lysing cells without the use of chemicals or detergents. Our approach is simple to integrate into microfluidic devices and compatible with high throughput single cell screening assays. While our method is limited in throughput by the upper rate at which monodisperse droplets can be generated and the Poisson loading that results in a majority of empty droplets, methods such as bubble-triggered droplet generation, geometrically mediated droplet breakup,^45,46^ and parallelization,^47,48^ can increase droplet generation rate markedly, while inertial ordering can massively reduce the number of empty droplets.^49^ The use of high voltages and conductive buffers should be assessed when working with voltage sensitive or heat sensitive proteins. In addition, lysozyme may not be compatible with all assays, and thus may be left out when necessary, but will also reduce lysis efficiency. We anticipate that our approach will provide an attractive alternative for applications that require cell lysis, but can be perturbed by the inclusion of common lysing agents, such as when characterizing binding or chemical activity of proteins, for screening and evolution applications. This method should also be valuable for lysing cells in droplets prior to mass-spectrometry analysis, which can be greatly hampered by the inclusion of common detergents. While we have demonstrated this approach with bacteria, we anticipate that it will prove equally effective for enhancing the lysis of other organisms, such as viruses, yeast, and mammalian cells.
